# A Two-state Model for the Diffusion of the A_2A_ Adenosine Receptor in Hippocampal Neurons: AGONIST-INDUCED SWITCH TO SLOW MOBILITY IS MODIFIED BY SYNAPSE-ASSOCIATED PROTEIN 102 (SAP102)*

**DOI:** 10.1074/jbc.M113.505685

**Published:** 2014-02-07

**Authors:** Patrick Thurner, Ingrid Gsandtner, Oliver Kudlacek, Daniel Choquet, Christian Nanoff, Michael Freissmuth, Jürgen Zezula

**Affiliations:** From the ‡ Institute for Pharmacology, Center for Physiology and Pharmacology, Medical University of Vienna, Währinger Str. 13a, 1090 Vienna, Austria and; the § Institut Interdisciplinaire de Neurosciences, CNRS UMR 5297, Université Bordeaux 2, 146 rue Léo Saignat, 33077 Bordeaux, France

**Keywords:** 7-Helix Receptor, Adenosine, Adenosine Receptor, Adenylate Cyclase (Adenylyl Cyclase), G Protein-coupled Receptor (GPCR), G Protein, Neuron

## Abstract

The A_2A_ receptor is a class A/rhodopsin-like G protein-coupled receptor. Coupling to its cognate protein, G_s_, occurs via restricted collision coupling and is contingent on the presence of cholesterol. Agonist activation slows diffusion of the A_2A_ adenosine receptor in the lipid bilayer. We explored the contribution of the hydrophobic core and of the extended C terminus by examining diffusion of quantum dot-labeled receptor variants in dissociated hippocampal neurons. Single particle tracking of the A_2A_ receptor(1–311), which lacks the last 101 residues, revealed that agonist-induced confinement was abolished and that the agonist-induced decrease in diffusivity was reduced substantially. A fragment comprising the SH3 domain and the guanylate kinase domain of synapse-associated protein 102 (SAP102) was identified as a candidate interactor that bound to the A_2A_ receptor C terminus. Complex formation between the A_2A_ receptor and SAP102 was verified by coimmunoprecipitation and by tracking its impact on receptor diffusion. An analysis of all trajectories by a hidden Markov model was consistent with two diffusion states where agonist activation reduced the transition between the two states and, thus, promoted the accumulation of the A_2A_ receptor in the compartment with slow mobility. Overexpression of SAP102 precluded the access of the A_2A_ receptor to a compartment with restricted mobility. In contrast, a mutated A_2A_ receptor (with ^383^DVELL^387^ replaced by RVRAA) was insensitive to the action of SAP102. These observations show that the hydrophobic core *per se* does not fully account for the agonist-promoted change in mobility of the A_2A_ receptor. The extended carboxyl terminus allows for regulatory input by scaffolding molecules such as SAP102.

## Teaser

**Background:** Agonist activation slows diffusion of the A_2A_ receptor in the lipid bilayer.

**Results:** In hippocampal neurons, the agonist-induced decrease in mobility was accounted for by both the hydrophobic receptor core and its extended C terminus, which recruited SAP102.

**Conclusion:** The observations are consistent with two diffusion states of the A_2A_ receptor in neurons.

**Significance:** SAP102 regulates access of the A_2A_ receptor to a compartment with restricted mobility.

## Introduction

Adenosine is the metabolite of last resort. Cell damage and hypoxia lead to its extracellular accumulation. As a consequence, four G protein-coupled receptors (termed A_1,_ A_2A_, A_2B_, and A_3_) are activated. The combined action of these four receptors is to orchestrate short- and long-term responses that further mitigate cell damage and activate tissue repair (1). This retaliatory metabolite concept can be exemplified by considering the role of the G_s_-coupled A_2A_ receptor in the vasculature, where short-term activation leads to vasodilation and increased blood supply, and long-term activation causes endothelial cell proliferation (2). In addition, adenosine is formed by the sequential action of ectonucleotidases from ATP that is stored in and released from synaptic vesicles (3). Thus, in the nervous system, adenosine acts as a neuromodulator. In the indirect pathway of the basal ganglia, for instance, volume transmission by adenosine engages striatal A_2A_ receptors and determines the set point for wired transmission (4). Accordingly, genetic deletion of A_2A_ receptors in the brain has complex phenotypic consequences, depending on whether the receptor is deleted globally or eliminated from specific regions (5). The psychomotor stimulant action of cocaine, for instance, is enhanced by ablation of the A_2A_ receptor in the striatopallidal projection neurons but blunted by elimination in the forebrain (6).

The A_2A_ adenosine receptor has several unique properties when compared with its closest relatives (*i.e.* the other adenosine receptors) and to the entire class of class A/rhodopsin-like GPCRs^3^ (7). Its C terminus is very long (122 amino acids) and, thus, provides docking sites for interacting proteins (8). It lacks the canonical palmitoylated cysteine residue, which is thought to anchor helix 8 in the membrane (9). It undergoes restricted collision coupling with its cognate G protein, G_s_ (10). The A_2A_ adenosine receptor can also stimulate mitogen-activated protein kinase/ERK by a cAMP-independent pathway (11, 12) that is contingent on recruitment of ARNO (the exchange factor for ARF6) (13). Cholesterol binds tightly to the A_2A_ receptor (14, 15), and this affects the ability of the receptor to recruit signaling molecules (16). We examined previously the diffusion mode of the A_2A_ receptor and an artificially palmitoylated version thereof by single particle tracking. These experiments indicated that the absence of the palmitoylated cysteine was required for restricted collision coupling. In addition, the experiment showed that lack of the C-terminal palmitoyl moiety promoted association of the agonist-activated A_2A_ receptor with detergent-resistant membranes and confinement in areas compatible in size with lipid rafts (17). In a parsimonious explanation, agonist-induced changes in the conformation of the heptahelical hydrophobic core determine the diffusive properties of the A_2A_ receptor by increasing its dwell time in cholesterol-rich lipid microdomains. Alternatively, the A_2A_ receptor may be tethered to additional molecules via its extended carboxyl terminus, and this may impinge on its diffusive properties. Here we examined the contribution of the C terminus by comparing the diffusion of a truncated but signaling-competent receptor with the full-length receptor in dissociated hippocampal neurons. The experiments showed that the hydrophobic core does not suffice to explain the diffusive properties of the receptor. The C terminus specifies access of the receptor to areas of low mobility by recruiting scaffolding molecules, one of which was identified as SAP102.

## EXPERIMENTAL PROCEDURES

### 

#### 

##### Expression Vectors

The following expression vectors were produced by standard methods in molecular biology: rat SAP102 (synapse-associated protein of 102 kDa, cDNA provided by Eckart Gundelfinger, Institute of Neurobiology, Magdeburg, Germany) fused at its N terminus to enhanced YFP (YFP-SAP102) and GST vectors (pGEX) coding for fusion proteins comprising rat SAP102. Human A_2A_ receptor with an N-terminal FLAG tag was mutated by QuikChange lightning site-directed mutagenesis (Stratagene, Carlsbad, CA) to replace ^382^DVELL^386^ of the distal carboxyl terminus with an RVRAA sequence. All constructs were verified by fluorescent DNA sequencing. The following constructs have been described previously (13, 18, 19): the full-length human A_2A_ receptor; the receptor comprising the codons for amino acids 1–311 (18), referred to as truncated A_2A_ receptor(1–311), tagged with an N-terminal FLAG epitope (13) or with a C-terminal enhanced cyan fluorescent protein (19); and an expression vector for the maltose-binding protein (MBP) fused to the carboxyl terminus of the A_2A_ receptor (MBP-A2A-c-tail) (13).

##### Culture of Hippocampal Neurons and Single Particle Tracking

Hippocampal neurons were prepared from 1- to 3-day-old rats. Papain-digested brain tissue (papain, 25 units/ml in Leibovitz L-15 medium containing 2 mm kynurenic acid) was dissociated by trituration. Cells were resuspended in culture medium (Neurobasal A, 2% B27, 1% heat-inactivated fetal calf serum, 0.4 mm glutamine, and 50 μm kynurenic acid) and plated on glass coverslips coated with poly-d-lysine. The culture medium contained 5-fluorouracil (20 μm) to inhibit cell proliferation. After 5 days, cells were transfected using Lipofectamine 2000 according to the instructions of the manufacturer (Invitrogen). Single particle tracking experiments were performed after 7 days in culture. Images of hippocampal neurons cotransfected with the A_2A_-receptor-CFP and YFP-SAP102 expression vectors were recorded with an epifluorescence microscope (Zeiss Axiovert 200 M) at ×63 magnification.

The single particle tracking experiments were performed as described previously in detail (17) for HEK293 cells. Briefly, transfected cells on coated glass coverslips were labeled with primary antibody recognizing the receptor FLAG epitope and the anti-mouse F(ab′)_2_-fragment-quantum dot conjugate. Quantum dot conjugates (Q-dots) were mixed with M2 anti-FLAG antibody at equimolar concentrations in Krebs-HEPES buffer (10 mm HEPES-NaOH (pH 7.5), 120 mm NaCl, 3 mm KCl, 2 mm MgCl_2_, 2 mm CaCl_2_, and 22 mm glucose) containing 3% casein for blocking of nonspecific binding sites. From this mixture, which contained quantum dots at a concentration of 0.5 μm and antibody at 0.5 μg/μl, aliquots were diluted (∼1:10^4^-1:10^5^) for labeling. After incubating for 20 min at 37 °C, coverslips were rinsed with Krebs-HEPES buffer, inserted into a microscopy chamber (buffer-filled volume of ∼50 μl), and transferred onto the heated stage of an Axiovert 135 M inverted epifluorescence microscope. Recordings were performed at 37° in the presence of adenosine deaminase (5 μg/ml) and, if indicated, of CGS21680 (10 μm). Optical stacks were acquired in Visiview 1.7.0 (Visitron Systems, Puchheim, Germany) as described previously (17) with the following modifications. The minimum length of a cognate trajectory was 50 frames. An intermission by quantum dot blinking of 10 frames duration was tolerated. Recordings with longer blinking events were dismissed, as were trajectories with out-of-focus events. Stacks were recorded at a rate of 50 frames/s, except when full frames were acquired (∼30 frames/s). To exclude spurious trajectories, cells were visualized by bright field illumination immediately after the fluorescence recordings.

##### Analysis of Quantum Dot Trajectories

Stack files were analyzed in MATLAB R2009a (MathWorks, Natick, MA) as described previously (17). The mean square displacement (MSD) of each particle was calculated using Equation 1,

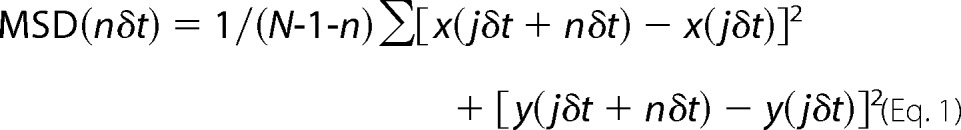
 where *n* is the time lag from start (number of frames), δ*t* is the duration of the optical frame in milliseconds, *N* is the total number of frames, and *j* is the index to Σ. Microscopic diffusion coefficients (*D*_initial_) were estimated by fitting the initial four data points of the mean square displacement (MSD_initial_) curve to an affine function (Equation 2),

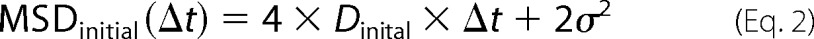
 where σ returns the *y* axis intercept of the linear regression.

The pool of *D*_initial_ values obtained in a given experiment (trajectories collected in an experimental setting as defined by the construct(s) used for transfection and by the presence of the receptor agonist during recordings and typically amounting to more than 200 values each) was subjected to a cumulative frequency distribution analysis and depicted as bar chart histogram.

In addition, the diffusion mode of each individual trajectory was examined for deviation from random walk (*i.e.* Brownian) motion, where the cumulative mean square displacement of the particle extends linearly with time. The classification of tracks as following directed or confined diffusion relied on the goodness of fit when tracks were fitted to equations 3 and 4. A diffusion mode was categorized as “confined diffusion” if, according to Equation 3, the ratio of *D*_intial_ values over the maximum displacement between any two points of the trajectory (*R*^2^) was found to be larger than a predefined threshold (log_10_ (ψ), set to a conservative value of 10).

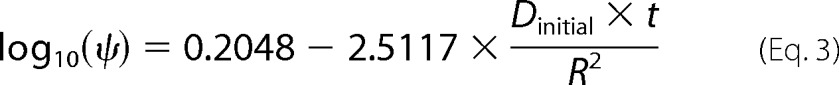


Trajectories displaying a log_10_ (ψ) greater than the threshold were then fitted by the trust region method to estimate the range covered by a particle traveling in confined diffusion mode. The criterion for “directed diffusion” was met if MSD data were fitted with appreciable goodness to a second-degree polynomial function (Equation 4).

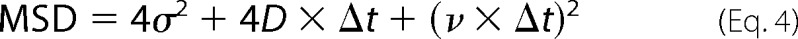


Particles were classified as immobile if none of the derived *D*_initial_ values exceeded a preset value of 0.0075 μm^2^/s. Assigning particle tracks to non-random motion must inevitably be ambiguous because any given track or major segments of it may occur with residual probability by random movement (20). However, the automated assignment prevents a significant bias in quantifying the relative size of the four groups of tracks.

##### Estimation of Receptor Diffusivity According to a Hidden Markov Model

Pooled trajectories were fitted to a two-state hidden Markov process (21). Maximum likelihood optimization with a Metropolis-Hastings algorithm returns estimates of the underlying parameters. The set of parameter estimates (θ) comprises the diffusion coefficients characteristic of states 1 and 2 (D1 and D2) and the probabilities of the particle transitioning between state 1 and state 2 (p12 and p21). We implemented, in MATLAB R2011a, the algorithms devised by Das *et al.* (21). For an estimation of forward-backward variables, we made use of the compatible fwdback.m program file, which replaced algorithms 2 and 4 in Ref. 21. The likelihood of the estimate of θ is proportional to the joint probability density function of all the tracks from one experimental group as given by

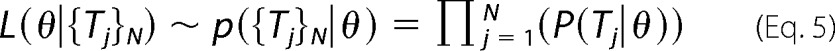
 where *T***_j_** is the observed *j*th trajectory, *N* is the number of all trajectories per group, and *P* is the probability of a given trajectory to obey the parameters θ. The burn-in period consisted of 2 × 10^3^ iterations, and the full routine was completed after 2 × 10^4^ iterations. The maximum likelihood estimate for each of the four parameters was derived from a histogram of accepted parameters after burn-in. The coefficient of variation (*CV*) was calculated in percent of the estimate according to Equation 6,

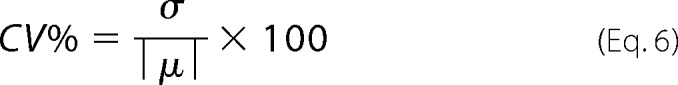
 where σ is the standard deviation and μ is the mean for the parameter distribution obtained from the optimization procedure. *CV* values were typically below 1% except where indicated. Optimized parameter estimates were further used to calculate the distribution between mobility states 1 and 2 (π_1_ and π_2_, Equation 7).

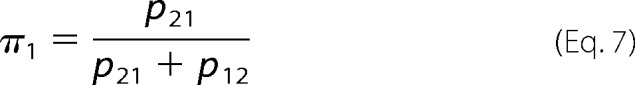
 Overall mobility of the receptor in an experiment is captured by the effective diffusion coefficient D_eff_ (Equation 8).

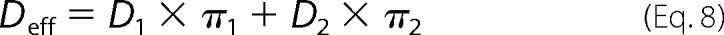


##### Pulldown Assays and Immunoprecipitation of the FLAG-tagged A_2A_ Receptor

SAP102 protein domains were assayed for interaction with the C terminus of the A_2A_ receptor using recombinant fusion proteins as in Ref. 13. MBP-A_2A_-CT, a fusion protein comprising the C terminus of the A_2A_ receptor, and individual GST-fused SAP102 domains were produced in *Escherichia coli* and purified. MBP-A_2A_-CT (8 μg) was incubated with the individual GST-fused SAP domains (8 μg each) for 30 min at 4 °C in a volume of 0.1 ml containing 1% Nonidet P-40. Complexes were immobilized by the addition of 50 μl GSH-Sepharose (1:1 pre-equilibrated slurry) and analyzed following SDS-gel electrophoresis by immunoblotting. Alternatively, complexes were retrieved via MBP by binding to amylose resin (13).

HEK293 cells were transiently transfected with plasmids coding for the FLAG-tagged wild-type and mutant versions of the A_2A_ receptor and YFP-SAP102. Immunoprecipitation was carried out from cell lysates solubilized in 1% Nonidet P-40 as outlined in Ref. 13. Proteins separated on an SDS-gel and blotted on nitrocellulose were detected with an antibody directed against the A_2A_ receptor (Merck-Millipore, catalog no. 7F6-G5-A2) and FLAG-specific antibodies (M2 mouse monoclonal from Sigma or rabbit polyclonal SC-807 from Santa Cruz Biotechnology).

##### Determination of Cellular cAMP and Phosphorylated ERK in HEK293 Cells

Cyclic AMP formation was assayed in HEK293 cells prelabeled with [^3^H]adenine (22). The phosphorylation of the ERK p44 and p42 isoforms (ERK 1/2) in HEK293 cells was determined after overnight serum deprivation as described in Ref. 18.

## RESULTS

### 

#### 

##### Single Particle Tracking of the Wild-type A_2A_ Receptor and of a C-terminally Truncated Version

**FIGURE 1.**
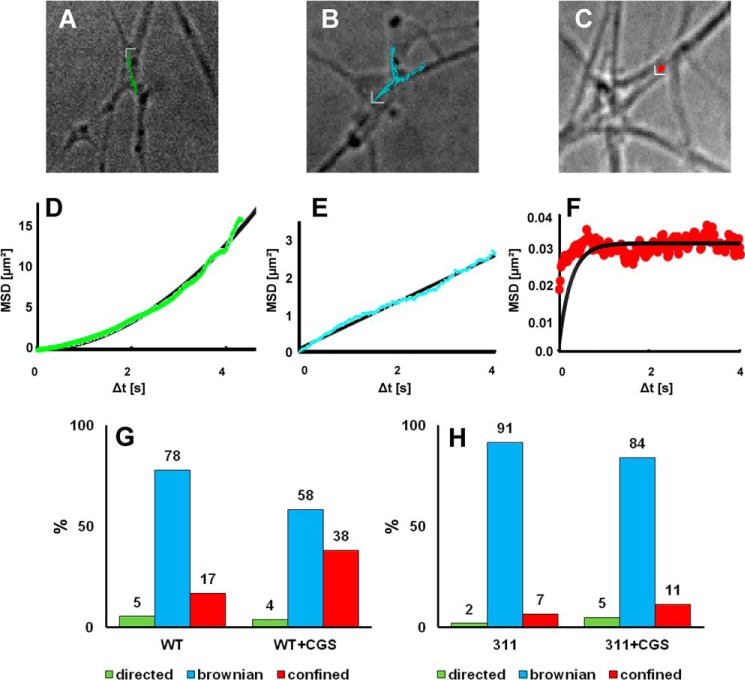
**Diffusion modes of quantum dot-labeled A_2A_ receptors on hippocampal neurons.** *A–C*, representative trajectories of the FLAG-tagged wild-type A_2A_ receptor were recorded as outlined under “Experimental Procedures” and projected onto the bright field image of the corresponding neuron. The *scale bars* adjacent to the trajectories define a square of 0.8 × 0.8 μm^2^. *D–F*, representative MSD plots highlighting three different diffusion modes: directed (*D*, *green circles*), Brownian/random walk (*E*, *cyan circles*), and confined (*F*, *red circles*). The *lines* were generated by fitting the data points to a second order polynomial (*D*), a straight line (*E*), and Equation 5 (*E*). *G* and *H*, the relative contribution of each diffusion mode (*green*, directed; *cyan*, Brownian; *red*, confined) was quantified by recording 614 and 890 trajectories for the wild-type A_2A_ receptor (*WT*, *G*) and 105 and 211 trajectories for the truncated A_2A_ receptor(1–311) (*311*, *H*), in the absence and presence of the agonist CGS21680 (+*CGS*, 10 μm), respectively.

Agonist-induced changes in the mobility of the A_2A_ receptor may be mediated by binding of cholesterol to the hydrophobic core (14, 15) or by tethering via the extended C terminus. We addressed this directly by measuring the trajectories of the quantum dot-labeled wild-type (full-length) receptor and of a C-terminally truncated version in neurite extensions of dissociated hippocampal neurons. This A_2A_ receptor(1–311) comprises helix 8 but lacks 101 residues of the extended C terminus. It represents the minimum version of a functional A_2A_ receptor because it activates both G_s_-dependent cAMP accumulation and G_s_-independent phosphorylation of ERK (18). Fig. 1 shows representative overlays of neurite extensions and quantum dot trajectories. Four possible macroscopic diffusion modes are conceivable, namely directed (*cf.* Fig. 1, *A* and *D*), Brownian/random walk (*cf.* Fig. 1, *B* and *E*), restricted, and immobile. It is difficult to unambiguously define particles as completely immobile (23). Hence, we pooled restricted and immobile trajectories and categorized these as confined diffusion mode (*cf.* Fig. 1, *C* and *F*). The number of directed diffusion events was low in all experiments (*cf*. Fig. 1, *H* and *G*, *green bars*). Its proportion was neither affected in a statistically significant manner by agonist stimulation nor upon overexpression of SAP102 (see below). The apparent velocity (mean ± S.D. = 0.84 ± 0.089 μm/s) was also comparable under all conditions. Previously, directed motion was not detected in HEK293 cells expressing wild-type and mutated versions of the A_2A_ receptor (17). We did not explore the underlying basis for directed motion. However, it has been observed repeatedly with several receptors in neurons and has been attributed to transport events (24, 25). Consistent with our earlier findings (17), we observed that stimulation by the agonist CGS21680 resulted in a marked increase in the confinement of the wild-type A_2A_ receptor (from 17 to 38%, Fig. 1*G*). In contrast, truncation greatly reduced confinement (Fig. 1*H*). We also extracted the radii of the confinement area from the exponential fit (Fig. 1*E*). These areas were comparable in size (mean ± S.D. = 300 ± 45 nm) regardless of the receptor variant examined and of the experimental conditions.

**FIGURE 2.**
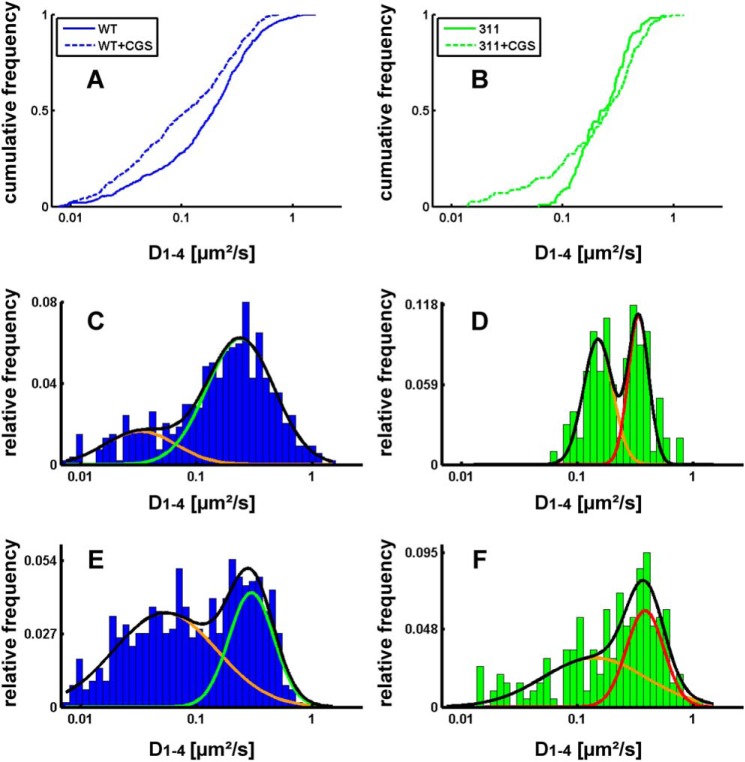
**Cumulative distribution frequency (*A* and *B*) and normalized histograms (*C–F*) of the diffusivity of the wild-type A_2A_ receptor (*A*, *C*, and *E*) and A_2A_ receptor(1–311) (*B*, *D*, and *F*).** Diffusivity was calculated from a linear fit to the mean square displacement of the first four recorded values (*D_1–4_*) as outlined under “Experimental Procedures.” The cumulative distribution frequency is on the basis of 539 and 625 trajectories recorded for the wild-type A_2A_ receptor and 102 and 200 recorded for the truncated A_2A_ receptor(1–311) (*311*) in the absence (*solid lines*) and presence of 10 μm CGS21680 (*CGS*) (*dashed lines*), respectively. Recordings of immobile particles (D_1–4_ <0.0075 μm^2^/sec) were excluded from the analysis. All distributions differ in a statistically significant manner (Kolmogorov-Smirnov test, in all instances *p* < 0.01). In *C–F*, the data were plotted as *histograms* to highlight the difference in the diffusivity of the wild-type (*C*) and the truncated A_2A_ receptor(1–311) (*D*) in the basal, inactive state (*C* and *D*) and the shift produced by agonist stimulation (*E* and *F*) of the wild-type receptor (*cf. C* and *E*) and the truncated version (*cf. D* and *F*). The *black lines* represent the fit assuming the presence of two Gaussian distributions. The individual populations are indicated by the *colored lines*.

We also examined the diffusivity (*i.e.* the area covered per time) and extracted the diffusion coefficients (D1–4) from the plots of mean square displacement over time. The analysis of the cumulative frequency distribution showed that, under basal conditions, there was a large and statistically significant difference (*p* = 0.000667 estimated by Kolmogorov-Smirnov test) in the distribution of microscopic diffusion coefficients between the wild-type receptor (Fig. 2*A*, *solid line*) and the truncated A_2A_ receptor(1–311) (Fig. 2*B*, *solid line*). For the wild-type receptor, it is readily evident that the distribution of diffusion coefficients was not accounted for by a homogeneous population of diffusing species. In fact, replotting the diffusion coefficients as histograms revealed a distribution that was adequately fitted by the sum of two Gaussian curves (Fig. 2*C*). The analysis of the histogram also revealed at least two populations of diffusive species in the truncated A_2A_ receptor(1–311) (Fig. 2*D*). Agonist stimulation shifted the cumulative frequency distribution of the wild-type A_2A_ receptor to the left (Fig. 2*A*, *dashed line*). The agonist-induced shift in the distribution is readily appreciated from the histograms. Agonist stimulation increased, more than 2-fold, the relative proportion of receptor species that diffused slowly (*cf*. Fig. 2, *C* and *E*). Agonist stimulation also affected the diffusion of the truncated A_2A_ receptor(1–311), resulting in the appearance of slowly migrating species (Fig. 2*B*, *dashed line*). However, on average, these slowly moving receptors diffused more rapidly than the wild-type receptor, and their relative proportion was lower (*cf.* Fig. 2, *E* and *F*).

##### Interaction between the A_2A_ Receptor and SAP102 in Vitro

**FIGURE 3.**
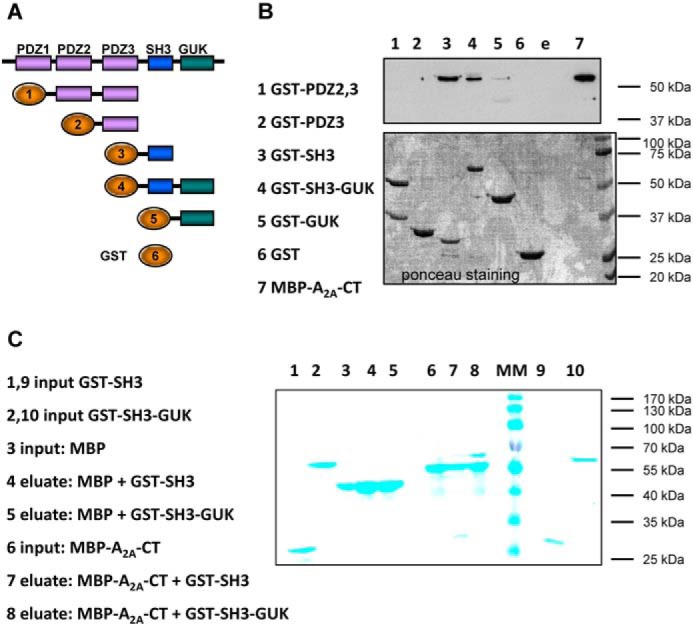
**Binding of the C terminus of the A_2A_ receptor fused to MBP to fusion proteins comprising the domains of SAP102 and GST.** *A*, schematic of the domain structure of SAP102 and of the GST fusion proteins employed. The numbers correspond to the lanes of the gel shown in *B. B*, purified fusion protein comprising MBP and the carboxyl terminus of the A_2A_ receptor (*MBP-A_2A_-CT*, 8 μg) was incubated in the presence of purified GST fusion proteins (8 μg each) comprising the indicated domains of SAP102 (*lanes 2–5*) or of GST (8 μg, *lane 6*) for 30 min at 4 °C in 0.1 ml of buffer. Proteins bound to GSH-Sepharose were released by denaturation. Aliquots corresponding to 25% of the eluate and MBP-A_2A_-CT (5% of the input, *lane 7*) were loaded onto a polyacrylamide gel, resolved electrophoretically, and transferred to nitrocellulose membranes that were probed with an antiserum directed against MBP (*top panel*). The *bottom panel s*hows the corresponding nitrocellulose stained with Ponceau S to visualize the GST fusion proteins recovered in the eluate. *C*, MBP and the fusion protein MBP-A_2A_-CT (each at 8 μg) were incubated with fusion proteins comprising GST and the SH3 domain (*GST-SH3*) or the combined SH3-GUK domain (*GST-SH3-GUK*) of SAP102 (each at 8 μg) as described in *B*. The proteins were recovered by binding to amylose resin and released by denaturation. Aliquots corresponding to 10% of the input and 10% (MBP-A_2A_-CT) or 20% of the eluate (MBP) were applied onto and resolved on a polyacrylamide gel. Proteins were visualized by staining with Coomassie Blue. Shown are representative experiments that were reproduced twice.

The observations summarized in Figs. 1 and 2 suggested that the hydrophobic core *per se* sufficed to account for the decreased mobility of the agonist-occupied receptor. However, it was also readily evident that the C terminus played a major role in specifying the mobility of the receptor. Accordingly, we searched for candidate mechanisms. We identified a fragment comprising the GUK domain and the adjacent SH3 domain of the postsynaptic protein SAP102 as a possible interaction partner in a yeast two-hybrid interaction screen using the C terminus of the A_2A_ adenosine receptor as bait. The interaction of this fragment of SAP102 with the C terminus induced the same level of β-galactosidase activity as ARNO/cytohesin 2 and USP-4 or (data not shown and Refs. 13, 19). The direct physical interaction between the two protein fragments was confirmed *in vitro* by pulldown assays. Fusion proteins were engineered to comprise GST and the domains of SAP102 (outlined in Fig. 3*A*), whereas the A_2A_ receptor C terminus was fused to the MBP to generate the construct A_2A_R_Ct_-MBP. The purified fusion proteins were combined, and complex formation was assessed by immobilization on GSH-Sepharose. The A_2A_R_Ct_-MBP fusion protein was retrieved with GST-SH3 (Fig. 3*B*, *lane 3*), GST-SH3/GUK (Fig. 3*B*, *lane 4*), and GST-GUK (Fig. 3*B*, *lane 5*) but not with GST-PDZ domains (Fig. 3*B*, *lanes 1* and *2*) or GST alone (Fig. 3*B*, *lane 6*). Similarly, GST-SH3 and GST-SH3-GUK were pulled down with the C terminus of the A_2A_ receptor fused to MBP but not with MBP (Fig. 3*C*). These experiments confirmed the finding of the yeast two-hybrid approach, ruled out that the interaction required an intermediary protein, and assigned the region of interaction to the C-terminal part of SAP102.

##### Coexpression of the A_2A_ Receptor and SAP102 in Mammalian Cells

**FIGURE 4.**
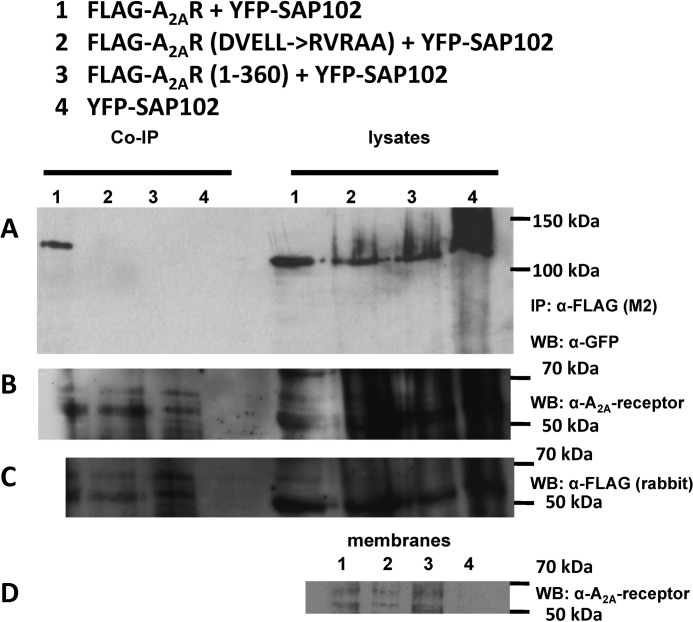
**Coimmunoprecipitation of SAP102 with wild-type and mutated versions of the A_2A_ receptor.** *A*, detergent lysates were prepared from HEK293 cells (1 × 10^6^ cells containing 0.53 ± 0.08, 0.45 ± 0.09, and 0.50 ± 0.10_._ pmol receptors for wild-type A_2A_ receptor, A_2A_ receptor-RVRAA, and A_2A_ receptor(1–360), respectively, as assessed by binding of [^3^H]ZM241385) coexpressing YFP-tagged SAP102 with FLAG-tagged versions of the wild-type A_2A_ receptor (*lane 1*), the A_2A_ receptor-^383^RVRAA^387^, a version with a mutated DVELL motif (*lane 2*), and the truncated A_2A_ receptor(1–360) (*lane 3*) or cotransfected with an empty plasmid (*lane 4*) and incubated with anti-FLAG M2 affinity matrix. Bound proteins were released by heat denaturation in SDS-containing reducing sample buffer. The immunoprecipitated material was electrophoretically resolved together with an aliquot (20 μg) of the corresponding lysate, transferred to nitrocellulose, and probed with an antiserum directed against GFP (*A*). *IP*, immunoprecipitation; *WB*, Western blot. The nitrocellulose blots were also probed with a monoclonal antibody directed against the A_2A_ receptor (epitope in the third intracellular loop, *B*) or a rabbit polyclonal antiserum raised against the FLAG epitope (*C*). Note that the immunoreactive bands corresponding to the various glycosylated forms of the A_2A_ receptor can only be detected in the immunoprecipitate but not in the lysates. Membranes (20 μg) prepared from HEK293 cells coexpressing YFP-tagged SAP102 with FLAG-tagged versions of the wild-type A_2A_ receptor (*lane 1*), the A_2A_ receptor-^383^RVRAA^387^, a version with a mutated DVELL motif (*lane 2*), and the truncated A_2A_ receptor(1–360) (*lane 3*) or cotransfected with an empty plasmid (*lane 4*) were resolved electrophoretically, transferred to nitrocellulose, and probed with the monoclonal antibody directed against the A_2A_ receptor (*D*).

**FIGURE 5.**
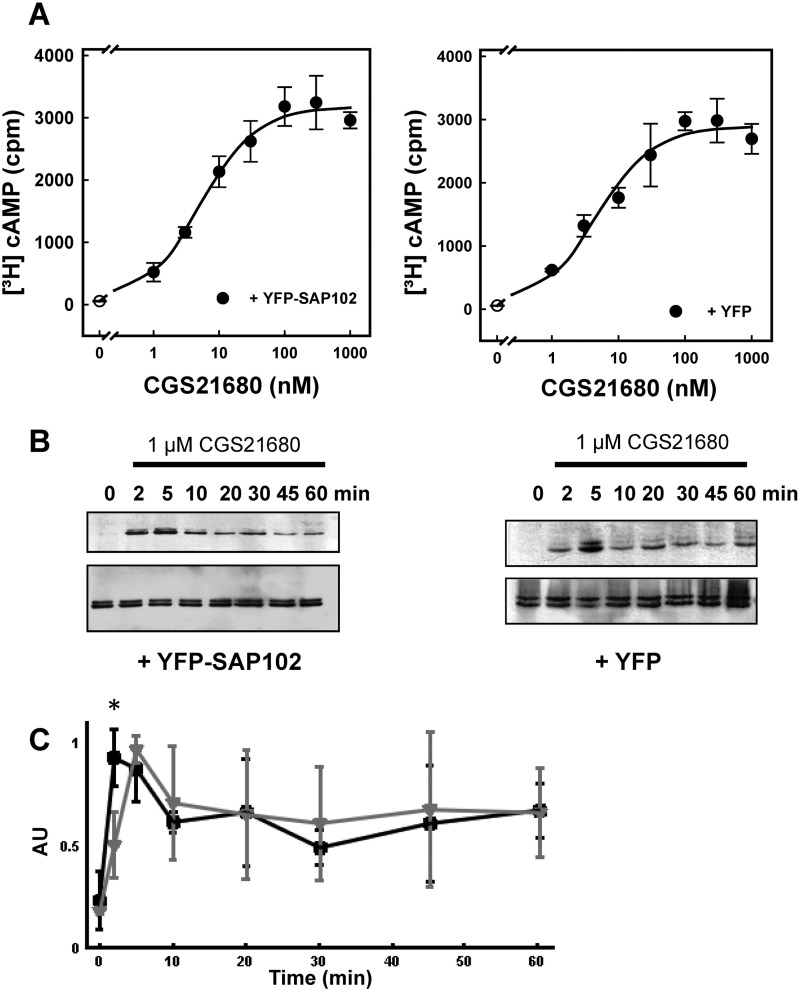
**Concentration-response curves for A_2A_ receptor-dependent [^3^H]cAMP accumulation (*A* and *B*) and time course of A_2A_ receptor-stimulated ERK phosphorylation in HEK293 cells in the absence and presence of coexpressed SAP102 (*B* and *C*).** HEK293 cells were transiently transfected with plasmids encoding the A_2A_ receptor and either YFP-SAP (*left panels*) or YFP (*right panels*). *A*, cells (3 × 10^5^/well) were labeled metabolically with [^3^H]adenine. After 16 h, fresh medium containing the indicated concentrations of CGS21680 was added, and cAMP production was stimulated for 20 min. Data are the means ± S.D. from three experiments. *B*, cells (3 × 10^5^/well) were serum-starved for 16 h and subsequently stimulated with 1 μm CGS21680 for the indicated time intervals. Aliquots of cellular lysates (20 μg) were applied to SDS-polyacrylamide gels. After electrophoretic resolution and transfer to nitrocellulose, the level of active ERK1/2 was assessed by immunoblotting with an antiserum recognizing the dually phosphorylated active enzyme (p-ERK) (*top blots*) or an antiserum against holo-ERK1/2 (*bottom blots*) as loading control. *C*, the extent of ERK phosphorylation from three independent experiments carried out as shown in *B* was analyzed by densitometry. *AU*, arbitrary units. Data are means ± S.D. *Gray triangles* and *black squares* indicate the response seen in cells coexpressing YFP and SAP102, respectively. The initial response (*i.e.* ERK phosphorylation after 2 min) was significantly higher (*, *p* = 0.024, unpaired Student's *t* test) in the presence of SAP102.

The observations summarized above provide compelling evidence that the carboxyl termini of the A_2A_ receptor and SAP102 can interact. We verified that the A_2A_ receptor also formed a complex with SAP102 *in vivo* by coexpressing a FLAG-tagged version of the A_2A_ receptor and a GFP-tagged version of SAP102 in HEK293 cells. This allowed for visualizing the proteins at the cell surface (not shown, see also Fig. 5). HEK 293 cells were employed that expressed the wild-type and mutated versions of the A_2A_ receptor at levels commensurate with physiological levels (*i.e.* at about 0.5 pmol/mg, as determined in binding assays using the antagonist radioligand [^3^H]ZM241385). At these expression levels, it was neither possible to detect the A_2A_ receptors reliably in the detergent lysates with an monoclonal antibody directed against the third intracellular loop A_2A_ receptor (Fig. 4*B*) nor with an anti-FLAG antibody (Fig. 4*C*). However, mutant and wild-type receptors were detectable in membranes prepared from these cells (Fig. 4*D*).

Complexes were recovered from detergent lysates with anti-FLAG antibodies immobilized on Sepharose beads, and the eluate was analyzed by immunoblotting (Fig. 4*A*). Immunoreactive material with a molecular weight corresponding to a SAP102-GFP fusion protein was recovered with an antibody directed against GFP if cells expressed the FLAG-tagged A_2A_ receptor and SAP102-GFP (Fig. 4*A*, *Co-IP lane 1*). Only trace levels of SAP102 were coimmunoprecipitated when coexpressed in cells with a truncated version of the A_2A_ receptor lacking the last 52 amino acids (A_2A_R1–360, Fig. 4*A*, *Co-IP lane 3*) or in mock immunoprecipitations; *i.e.* antibody incubated with lysates from cells transfected with an empty plasmid rather than with the receptor-encoding plasmid (Fig. 4*A*, *Co-IP lane 4*). The lysates contained equivalent amounts of GFP-tagged SAP102A (Fig. 4*A*, *lysate lanes 1–4*). A protein of the same molecular weight was detected in the Western blot analysis when a commercially available antibody directed against SAP102 directly was used (not shown). When different mammalian orthologs of the A_2A_ receptor are examined, it is evident that there is only a limited conservation in sequence over the last 50–53 residues (7). The divergence includes differences in the overall length of the distal portion and several positions of charge reversal. Of the candidate conserved elements, we identified a ^383^DVELL^387^ motif (the numbering refers to the human ortholog) that, when mutated to RVRAA, abrogated the interaction of the A_2A_ receptor with SAP102 (Fig. 4*A*, *Co-IP lane 2*). We verified that the immunoprecipitates contained comparable levels of receptor. Both the antibody against the A_2A_ receptor (Fig. 4*A*, *Co-IP lanes 1–3* in the *lower blot*) and the FLAG epitope (not shown) detected several bands in the range of 50–60 kDa in the immunoprecipitates from lysates containing A_2A_ receptors but not in the immunoprecipitate from mock-transfected cells (Fig. 4*A*, *Co-IP lane 4*). We showed previously that these bands corresponded to the differently glycosylated forms of the A_2A_ receptor (26). As an additional control of specificity, we used the HA-tagged A_1_ receptor, which failed to interact with SAP102 (not shown).

The A_2A_ receptor is a prototypical G_s_/G_olf_-coupled receptor and, thus, elevates cAMP levels. In addition, it stimulates ERK phosphorylation via a G_s_-independent pathway (11, 12). Accordingly, we tested whether coexpression of SAP102 affected the A_2A_ receptor-generated signal by comparing to the response of control cells that coexpressed YFP. It is evident from Fig. 5*A* that the presence of SAP102 did not affect the ability of the agonist to increase cAMP. The concentration response curves were virtually superimposable (EC_50_ = 4.7 ± 1.3 and 5.3 ± 0.9 nm; maximum response E_max_ = 2838 ± 186 and 3122 ± 132 cpm; in the presence and absence of SAP102, respectively; *n* = 3). In contrast, coexpression SAP102 had a statistically significant effect on agonist-mediated stimulation of ERK1/2. There was a statistically significant increase in the time to reach peak ERK phosphorylation in the presence of SAP102 (Fig. 5, *B* and *C*). We also verified that mutation of the ^383^DVELL^387^ motif in the C terminus did not affect the ability of the resulting A_2A_ receptor-^383^RVRAA^387^ to raise cAMP levels in response to agonist stimulation (EC_50_ for CGS21680 = 7.3 ± 4.6 nm; E_max_ = 2869 ± 389 cpm).

**FIGURE 6.**
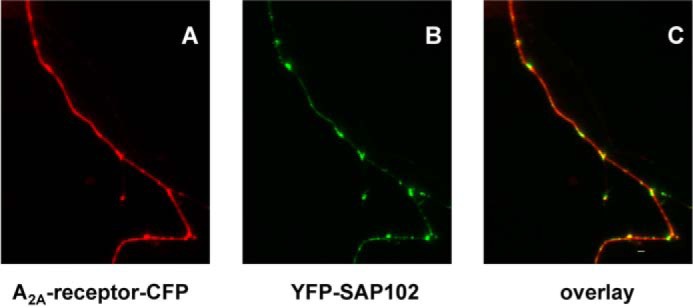
**Colocalization of A_2A_ receptors and SAP102 in hippocampal neurons.** Cultures of dissociated rat hippocampal neurons were transfected with plasmids coding for the CFP-tagged A_2A_ receptor (*A_2A_-receptor-CFP*) and YFP-tagged SAP (*YFP-SAP102*). Images were captured in both the CFP (*A*, *red pseudocolor*) and the YFP channel (*B*, *green pseudocolor*), noise-corrected, and converted into an 8-bit RGB image. An overlay image (*C*, *red*, *green*, and *yellow pseudocolors*; *scale bar* = 19 μm) was generated in MATLAB by combining the two channels. Regions with both proteins appear in *yellow pseudocolor*. Data are representative of >10 neurons in three independent transfections.

Interaction of SAP102 with the A_2A_ receptor is predicted to enhance local confinement of the receptor and to compartmentalize the receptor-generated signal. However, the A_2A_ receptor is known to undergo restricted collision coupling (10, 17) and to be, *per se*, subject to local confinement in HEK293 cells (17). Hence, it is not surprising that methods that rely on global readout of the response, such as cAMP accumulation (Fig. 5*A*), fail to detect any major effects induced by coexpression of SAP102. Finally, SAP102 is of particular relevance in organizing signaling complexes in neurons. On the basis of these considerations, we resorted to coexpressing the fluorophore-tagged versions of the A_2A_ receptor and SAP102 in neurons. The CFP-tagged A_2A_ receptor was distributed along the neurite extension and enriched in punctate structures (Fig. 6*A*). YFP-tagged SAP was inhomogeneously deposited along neurite extensions and concentrated in discrete regions (Fig. 6*B*). Image overlays showed that the A_2A_ receptor colocalized with SAP102 in these areas (Fig. 6*C*). It is worth noting that the A_2A_ receptor also accumulated in punctate structures in the absence of any overexpressed SAP102 (data not shown). The images shown in Fig. 5 and in subsequent figures were generated with an N-terminally tagged version of SAP102. Direct complex formation may also be visualized by FRET. However, in the N-terminally tagged SAP102, the (predicted) distance between the YFP moiety and the SH3-GUK domain (*i.e.* the interaction site with the A_2A_ receptor) is larger than the Förster distance of the YFP/CFP pair. Accordingly, YFP was also fused to the C terminus of SAP102. However, this protein was only expressed poorly (data not shown), thus precluding any further analysis.

##### Single Particle Tracking of a SAP102 Binding-deficient Mutant A_2A_ Receptor in Neurons

**FIGURE 7.**
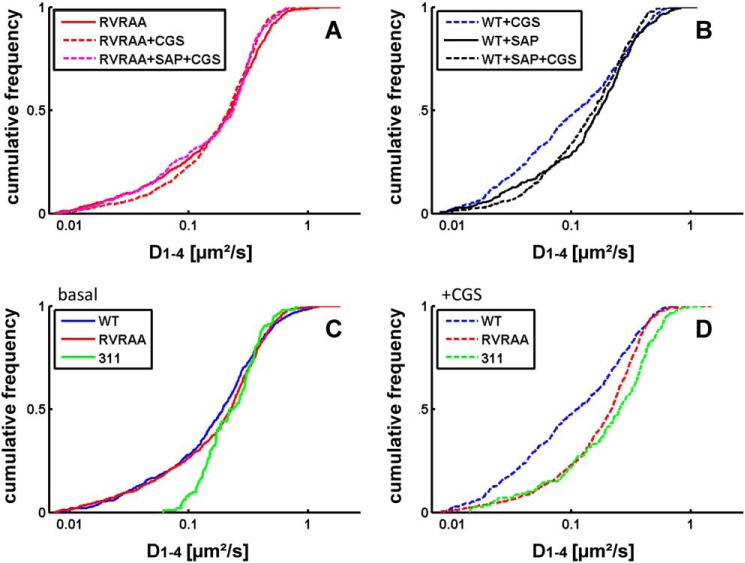
**Cumulative distribution frequency of the diffusivity of the A_2A_ receptor-^383^RVRAA^387^ (*A*) and the wild-type A_2A_ receptor (*B*) in the absence and presence of overexpressed SAP102.** Dissociated hippocampal neurons were transfected with plasmids driving the expression of A_2A_ receptor-^383^RVRAA^387^ (*A*, *red lines*) and A_2A_ receptor alone (*B*, *blue lines*) or in combination with a plasmid encoding YFP-tagged SAP102 (*magenta line* in *A* and *black line* in *B*). Receptors were labeled with quantum dots, and trajectories were recorded in the absence (*solid lines*) and presence of 10 μm CGS21680 (*dashed lines*). The cumulative frequency distribution obtained in the presence of SAP102 with A_2A_ receptor-^383^RVRAA^387^ (495 trajectories) overlapped with the other curves and was omitted for clarity. The curve for the agonist-stimulated wild-type A_2A_ receptor is the same as that shown in Fig. 2*B* and is included for comparison. In *A*, the cumulative distribution frequency is on the basis of 630 (basal), 885 (in the presence of 10 μm CGS21680), and 295 (with overexpression of SAP102 and stimulated with 10 μm CGS21680) trajectories. In *B*, 402 and 375 trajectories were recorded for the wild-type A_2A_ receptor in the presence of overexpressed SAP102 under basal and agonist-stimulated conditions, respectively. Recordings of immobile particles (*D_1–4_*, <0.0075 μm^2^/s) were excluded from the analysis. *C* and *D*, comparison of the cumulative distribution frequency under basal conditions (*C*) and after agonist stimulation (*D*) for the wild-type A_2A_ receptor (*blue line*, taken from Fig. 2*A*), A_2A_ receptor(1–311) (*green line*, taken from Fig. 2*B*), and A_2A_ receptor-^383^RVRAA^387^ (*red line*, taken from *A*).

Taken together, the data summarized in Figs. 4–6 predicted that diffusion of the mutant receptor with the charge reversal in the DVELL motif (^383^DVELL^387^ changed to ^383^RVRAA^387^) should not be affected by SAP102 levels. In contrast, overexpression of SAP102 was expected to alter the mobility of the agonist-activated, wild-type A_2A_ receptor. In the basal state, overexpression of SAP102 did not affect the diffusion properties of the examined receptors (shown for the wild-type receptor in Fig. 7*B*). In the absence of agonist, the distribution of diffusion coefficients observed for the A_2A_ receptor-^383^RVRAA^387^ (Fig. 7, *A* and *C*, *solid red lines*) did not differ from that of the wild-type A_2A_ receptor (Fig. 7*C*, *blue line*) in a statistically significant manner (*p* = 0.225, estimated by Kolmogorov-Smirnov test). Similarly, the cumulative frequency distribution of the truncated receptor A_2A_ receptor(1–311) (Fig. 7*C*, *green line*) differed in a significant manner from that of the mutant A_2A_ receptor-^383^RVRAA^387^ (*p* = 1.05 × 10^−6^). Most importantly, there was no statistically significant difference regardless of which condition was compared for the mutant A_2A_ receptor-^383^RVRAA^387^ (Fig. 7*A*; *p* = 0.103 for basal *versus* CGS21680; *p* = 0.281for basal *versus* CGS21680 in the presence of overexpress SAP102; *p* = 0.237 for the comparison of CGS21680 in the absence and presence of overexpressed SAP102). Accordingly, the distribution of the agonist-activated A_2A_ receptor-^383^RVRAA^387^ approached that of the agonist-activated truncated A_2A_ receptor(1–311) (*cf*. Fig. 7*C*, *green* and *red lines*). Nevertheless, their cumulative frequency distribution curves differed in a statistically significant manner (*p* = 0.00054) because there were more rapidly moving truncated A_2A_ receptors(1–311) than A_2A_ receptors-^383^RVRAA^387^. Finally, overexpression of SAP102 increased the mobility of the agonist-activated wild-type A_2A_-receptor (Fig. 7*B*, *dashed black line*). The cumulative frequency distributions of the agonist-liganded wild-type A_2A_ receptor differed in a statistically significant manner in the absence (Fig. 7*B*, *dashed blue line*) and presence (Fig. 7*B*, *dashed black line*) of overexpressed SAP102 (*p* = 1.84 × 10^−5^ by Kolmogorov-Smirnov test). Overexpression of SAP102 also abolished the agonist-induced increase in confinement (data not shown).

##### Analysis of Transition Rates by a Hidden Markov Model

**FIGURE 8.**
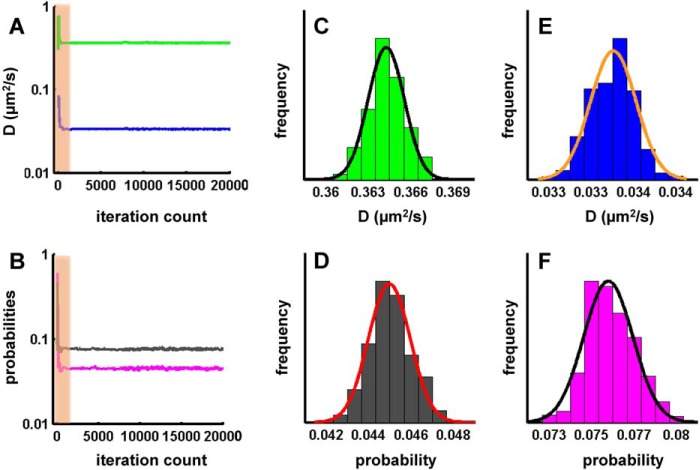
**Maximum likelihood optimization run for an analysis by a two-state hidden Markov model.** Optimization started with uniformly distributed random initial parameter estimates. *A* and *B*, representative plots of diffusion coefficients D1 (*A*, *green*) and D2 (*A*, *blue*) and for the probabilities p12 (*B*, *black*) and p21 (*B*, *magenta*) of A_2A_ receptor-^383^RVRAA^387^ *versus* the number of iterations. The *shaded area* represents the burn-in phase. *C–F*, uncertainty of parameter estimates for D1 (*C*), D2 (*D*), p12 (*E*), and p21 (*F*) fitted with a single Gaussian distribution.

**FIGURE 9.**
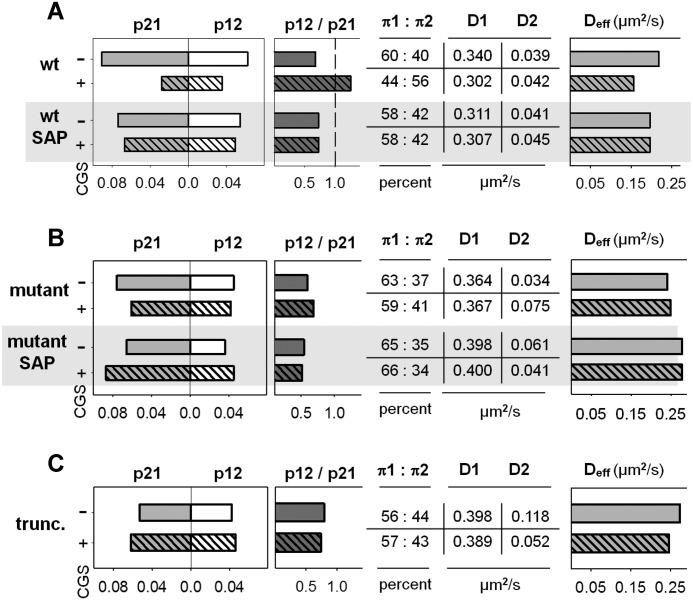
**Parameter estimates for the two diffusion states of the wild-type and mutant versions of the A_2A_ receptor obtained by a hidden Markov model.** For each individual condition shown, all trajectories were combined, and the resulting data points (25,000–250,000 displacements) were subjected simultaneously to optimization runs in a two-state hidden Markov model to extract parameter estimates as outlined under “Experimental Procedures.” Coefficient of variations were <1% for all parameters except for the A_2A_ receptor, where they amounted to 2–5%. *CGS*, CGS21680.

The cumulative frequency distributions shown in Figs. 2 and 7 are indicative of a minimum of two populations of receptors. This was also true for macroscopic diffusion if the small fraction of directed motion was ignored. In a simplified approach, it is therefore justified to assume an anisotropic membrane consisting of a slow and a fast region (which are reflected by the corresponding two states in the model). Thus, a protein exploring the membrane can be modeled as a sequence of movements in either domain. The diffusivity of the receptor, its dwell time in either state, and the probability of switching between the two states depends on its intrinsic properties (*e.g.* size or conformation) and on possible interactions (extrinsic). When tracking the A_2A_ receptor, the exact sequence of states it follows is hidden. If we consider the measured displacements as a reflection of either state, we can estimate the most likely sequence by optimizing the set of parameters (D1, D2, p21, and p12) to account for the observed displacements over time using a hidden Markov model. This approach allowed for testing whether changes in diffusion arose from alterations in state mobilities (*D*1 and *D*2), transition probabilities (*p*21 and *p*12), or both and for extracting equilibrium populations (π1 and π2), a pseudo K_D_* (π2/π1), and the effective diffusion coefficient of the system (*D*1 × π1 + *D*2 × π2). The power of the analysis relies on the fact that all data points are combined (*i.e.* 25,000–250,000/condition) and subjected simultaneously to a maximum likelihood fitting procedure. A typical optimization curve for an ensemble of trajectories is shown in Fig. 8. The graphic representation shows that the two-state hidden Markov model yielded stable parameters after a burn-in phase of <2000 iterations and adequately described the observational data set. An example of the uncertainty analysis is displayed for the calculated parameters *D*1 (Fig. 8*B*), *D*2 (Fig. 8*C*), *p*12 (Fig. 8*D*), and *p*21 (Fig. 8*E*) for the mutant A_2A_ receptor-^383^RVRAA^387^. These parameters were in accordance with values that can be estimated from the diffusivity analysis (*cf. e.g.* the histograms in Fig. 2 and the parameters summarized in Fig. 9). The rigorous quantification provided by the hidden Markov model runs generated the following insights. Under all conditions tested, the fast population of the wild-type A_2A_ receptor (Fig. 9*A*, *D1*) was consistently slower than that of the mutant A_2A_ receptor-^383^RVRAA^387^ (Fig. 9*B*, *D1*). As predicted on the basis of its inability to recruit SAP102, D1 (0.398 μm^2^/s, Fig. 9*C*) of the truncated A_2A_ receptor(1–311) was similar to that of the mutant A_2A_ receptor-^383^RVRAA^387^ (Fig. 9*B*). In contrast, the slower population (D2) was more homogenous with a mean of 0.048 ± 0.01 μm^2^/s. The only notable exception was the A_2A_ receptor(1–311) under basal conditions (Fig. 9*C*). Binding of the agonist to the wild-type A_2A_ receptor reduced the transition probability from state 1 to state 2 by a factor of 2 (*p*12, 0.062 → 0.035; Fig. 9*A*). Concomitantly, the probability of exiting state 2 declined by a factor of 3 (*p*21, 0.091 → 0.028; Fig. 9*A*). Thus, agonist stimulation enhanced the dwell time of the wild-type receptor in each state because transitions were less frequent and shifted the equilibrium distribution in favor of the slower population. This shift increased the pseudo K_D_* above 1 (Fig. 9*A*). There was also a minor change (by ∼10%) in the estimated diffusion coefficient in the fast population. The overall effect on the system is best appreciated by considering the ∼30% drop in the effective diffusion coefficient (0.218 μm^2^/s → 0.157 μm^2^/s in Fig. 9*A*).

Overexpression of SAP102 abolished the agonist-induced change in the effective diffusion coefficients (D_eff_, 0.197 μm^2^/s; Fig. 9*A*, *third* and *fourth rows*). Consistent with the diffusivity analysis (Fig. 7*B*), the effective diffusion coefficients observed after SAP overexpression were between that of the unliganded and that of the agonist-bound wild-type receptor (0.218 > 0.197 > 0.157 μm^2^/s). SAP overexpression modestly reduced the transition rates of the wild-type receptor in the basal state (*p*12, 0.062 → 0.054; *p*21, 0.091 → 0.074; *cf*. Fig. 9*A*, *first* and *third rows*), but the equilibrium distribution was virtually not affected (60:40 *versus* 58:42, Fig. 9*A*). Most importantly, after overexpression of SAP102, agonist binding only resulted in a barely detectable additional reduction in these transitions but did neither cause an inversion of the equilibrium populations nor a change in the pseudo K_D_* (*cf.* Fig. 9A, *p*12 and *p*21 in the *third* and *fourth rows*).

In contrast, agonist binding to the A_2A_ receptor-^383^RVRAA^387^ (Fig. 9*B*) and to the truncated A_2A_ receptor(1–311) (Fig. 9*C*) had a negligible effect on the transition rates *p*12 and *p*21 and, thus, on the resulting pseudo K_D_*. Accordingly, the effective diffusion coefficients were comparable in the absence and presence of agonist and were not affected by overexpression of SAP102. Consistent with the difference in cumulative distribution frequency (Fig. 7*C*), the slow population of the truncated A_2A_ receptor(1–311) was more than 2-fold faster (D2 = 0.118 μm^2^/s, Fig. 9*C*) in the basal state than in the average of all other experiments (D2 = 0.048 μm^2^/s). The agonist-induced broadening of the cumulative distribution frequency (Fig. 2*B*, *dashed line*) was reflected by a decline in *D*2 rather than by a change in the equilibrium distribution (Fig. 9*C*, π *:* π*2*) and, hence, in the pseudo K_D_*.

## DISCUSSION

Many G protein-coupled receptors have been shown to be attracted to specialized lipid microdomains in the agonist-bound state (27). This attraction to lipid rafts has been attributed to the presence of the palmitoyl moiety in helix 8 (28). Alternatively, the hydrophobic core may attract specific lipids by defining the thickness of the membrane in the immediate vicinity of the protein (28). Finally, membrane proteins are known to bind lipids tightly. In fact, bound cholesterol has been visualized in the crystal structures of several GPCRs, *i.e.* of rhodopsin (29), of the β_2_ (30) and the β_1_ adrenergic receptors (31), and of the A_2A_ receptor (14). These observations show that, in the agonist-bound state, the hydrophobic core of the A_2A_ receptor specifies an attraction to areas of slow mobility, but this is very much enhanced by the extended C terminus. This conclusion was reached on the basis of the following evidence. The A_2A_ receptor existed in a minimum of two mobility states in the membrane. This was evident from both the cumulative distribution frequency of individual trajectories and from the analysis of all trajectories by a hidden Markov model. Upon agonist binding to the A_2A_ receptor, transition between the two states was reduced. Accordingly, the receptor was shifted into the state that frequently visited the membrane compartment(s) with slow mobility. Truncation of the C terminus only reduced but did not eliminate the agonist-induced switch to slow mobility of the resulting A_2A_ receptor(1–311). Overexpression of SAP102 precluded access of the A_2A_ receptor to the compartment with restricted mobility. In contrast, the mutated A_2A_ receptor-^383^RVRAA^387^, which failed to recruit SAP102, was insensitive to the action of SAP102. Taken together, these findings define the contribution of the hydrophobic core, which undergoes the agonist-induced conformational change, and that of the C terminus, which allows for sampling the submembrane space for potential interactions. We rule out that our analysis is confounded by internalization events. Under the conditions employed, agonist-induced internalization was not observed, suggesting that it is a rare event within the first hour of agonist exposure (16).

The contribution of the extended C terminus is best appreciated by examining the distribution of diffusion coefficients in the truncated A_2A_ receptor(1–311) under basal conditions and comparing it to that of the wild-type receptor. In the absence of the agonist, there were two sharp peaks for the truncated A_2A_ receptor(1–311) (Fig. 2*D*). In contrast, the distribution was considerable broader for the wild-type receptor (Fig. 2*C*). This difference directly visualizes the impact of the C terminus on receptor diffusivity. It can be readily envisaged to result from the interaction of the extended C terminus with additional proteins, which lead to transient tethering of the receptor. One of these interactions was confirmed by examining the effect of SAP102. The analysis by the hidden Markov model showed that SAP102 regulated the access of the A_2A_ receptor to the compartment with restricted mobility. SAP102 (DLG3, disc large-3) is unique among the members of the PSD95 (postsynaptic density protein of 95 kDa) family of membrane-associated GUK proteins because it does not form homo-oligomers and does not have an N-terminal palmitoylation site or an L27 domain and, thus, relies on the SH3-Hook-GUK domain for membrane targeting (32). Finally, in contrast to PSD95, SAP102 is highly mobile and subject to rapid turnover in spines (32, 33). Interestingly, the region of SAP102 contacted by the A_2A_ receptor, the SH3-GUK domain, is also the region required for binding of mPINS (mammalian homolog of *Drosophila melanogaster* partner of inscrutable), which confers a G_i_-dependent regulation onto SAP102 via its GoLoco domain (34). This has interesting implications because the A_2A_ receptor can form heteromers with the G_i_-coupled D_2_-receptor. The interaction may result in mutual antagonism (35). This may be, in part, mediated by redistribution into membrane compartments that are less conducive to G protein coupling. In fact, D_2_/A_2A_ receptor heteromers differ in their mobility from D_2_ receptors (16). Although the interaction with SAP102 explains some diffusive properties of the A_2A_ receptor in hippocampal neurons, it is evident from our observations that there must be more interactors that bind to the C terminus because the diffusivity of the mutant receptor, which was deficient in SAP102 binding, also differed substantially from that of the truncated A_2A_ receptor(1–311). In fact, of the several proteins known to interact with the A_2A_ receptor (7, 8), of particular interest are those that provide a possible link to the cytoskeleton. α-Actinin has been shown previously to bind to the A_2A_ receptor (36). Our yeast-two hybrid interaction screen identified additional candidate interactors, one of which is non-erythrocytic α-spectrin (SPTAN-1).^4^

Our previous experiments defined the size of the confinement area in HEK293 cells (17). These differ modestly from our estimates obtained in hippocampal neurons. In HEK293 cells, agonist stimulation decreased the radius of confinement. This was not seen in hippocampal neurons. In the agonist-bound state, the receptor was confined to an area with a calculated radius of 300 nm, which was somewhat larger than the radius of confinement estimated in HEK293 cells (120 nm). This discrepancy can be rationalized if the compartmentalized lipid distribution in neurons is taken into account (37). In contrast to neurons, HEK 293 cells do not have any obvious polarized membranes. Regardless of the underlying cause, it is worth noting that the A_2A_ receptor is expressed endogenously in hippocampal neurons (38). Hence, our observations are of physiological significance for several reasons. Coexpression of SAP102 shifted the peak of ERK phosphorylation by some 3 min. Previous experiments showed that the truncated A_2A_ receptor(1–311) differed substantially from the wild-type receptor in its relative ability to activate cAMP accumulation and ERK phosphorylation (18). More recently, we showed that changes in the diffusion mode were directly linked to changes in the mode of adenylyl cyclase activation (restricted *versus* unrestricted collision coupling Ref. 17). Accumulation of a receptor in distinct membrane microcompartments is of particular interest in neurons because the location of the receptor determines the nature of the signal. In fact, compartmentalized signaling by the A_2A_ receptor can be observed in neuronal cells (39).

## Footnotes

4 M. Freissmuth, unpublished observation.

## Footnotes

3 The abbreviations used are:

GPCR: G protein-coupled receptor
MBP: maltose-binding protein
CGS21680: 3-[4-[2-[6-amino-9-[(2R,3R,4S,5S)-5-(ethycarbamoyl)3,4-dihydroxy-oxolan-2-yl]amino]ethyl]phenyl]propanoic acid
MSD: mean square displacement
GUK: guanylate kinase
CFP: cyan fluorescent protein.
